# The Condition for Generous Trust

**DOI:** 10.1371/journal.pone.0166437

**Published:** 2016-11-28

**Authors:** Obayashi Shinya, Inagaki Yusuke, Takikawa Hiroki

**Affiliations:** 1 Faculty of Economics, The University of Tokyo, Bunkyo, Tokyo, Japan; 2 Survey Science Center, The Institute of Statistical Mathematics, Tachikawa, Tokyo, Japan; 3 Frontier Research Institute for Interdisciplinary Sciences, Tohoku University, Sendai, Miyagi, Japan; University of Reading, UNITED KINGDOM

## Abstract

Trust has been considered the “cement” of a society and is much studied in sociology and other social sciences. Most studies, however, have neglected one important aspect of trust: it involves an act of forgiving and showing tolerance toward another’s failure. In this study, we refer to this concept as “generous trust” and examine the conditions under which generous trust becomes a more viable option when compared to other types of trust. We investigate two settings. First, we introduce two types of uncertainties: uncertainty as to whether trustees have the intention to cooperate, and uncertainty as to whether trustees have enough competence to accomplish the entrusted tasks. Second, we examine the manner in which trust functions in a broader social context, one that involves matching and commitment processes. Since we expect generosity or forgiveness to work differently in the matching and commitment processes, we must differentiate trust strategies into generous trust in the matching process and that in the commitment process. Our analytical strategy is two-fold. First, we analyze the “modified” trust game that incorporates the two types of uncertainties without the matching process. This simplified setting enables us to derive mathematical results using game theory, thereby giving basic insight into the trust mechanism. Second, we investigate socially embedded trust relationships in contexts involving the matching and commitment processes, using agent-based simulation. Results show that uncertainty about partner’s intention and competence makes generous trust a viable option. In contrast, too much uncertainty undermines the possibility of generous trust. Furthermore, a strategy that is too generous cannot stand alone. Generosity should be accompanied with moderate punishment. As for socially embedded trust relationships, generosity functions differently in the matching process versus the commitment process. Indeed, these two types of generous trust coexist, and their coexistence enables a society to function well.

## Introduction

In general, when one confronts a botched situation, one of the most effective ways to maintain existing relationships is to forgive the person who has erred. Everyone commits mistakes in social relationships such as friendships, business relationships, and romantic relationships. Some mistakes do not occur because of malicious intent but rather carelessness or incompetence. When we trust those who have committed mistakes and expect this mistake to be corrected, we forgive them in order to continue the relationship. For example, in a business relationship, if there is a delay in a delivery which might lead to profit loss, the client may tolerate the contractor especially if they are in a long-term trust relationship.

Previous studies, however, formulate trust relationship in a rather different way. Trust is considered to be a part of behavioral strategies that involves breaking off committed relationships when the partner commits a betrayal or makes a mistake (e.g., [1–3]). In this formulation, trust is inflexible. However, this approach does not adequately consider the aspect of trust that functions as forgiveness. Past research has often ignored various causes of mistakes and instead, focused on malicious intention because they adopt a perfect rationality framework. On the other hand, in real life and experimental settings, it is found that trust relationships can be maintained in spite of recipients’ defect even when punishment is not effective (e.g., Berg [4]). Thus previous works do not fully capture the phenomena related to trust and forgiveness. In fact, people do not always act in a rational manner and sometimes fail to cooperate with others unintentionally. In that case, forgiveness is common. Therefore, in oder to fully understand the trust relationship, we have to take account of the behavioral aspect which is found in the real life and the experimental settings.

In this study, we propose an alternative concept of trust called “generous trust” to capture the above-mentioned and often overlooked aspect of trust. Generous trust is the trust that functions as tolerance or forgiveness of a partner’s mistake, especially in cases where the mistake occurs by accident. This enables two parties to maintain a trust relationship.

Some sociologists do characterize the trust relationship in a similar manner. For example, Uzzi [5] reports that trust relationship enables parties to interpret their partner’s intention in a favorable way and are thus adequately equipped for dealing with unforseen conflict. Even in economic transactions, if two parties trust each other, a problem caused by one party does not immediately trigger the end of the relationship. Rather, the problem is not taken as a sign of betrayal but interpreted as a recoverable error and the relationship is maintained. Montgomery [6] formalizes this process by setting up two contrasting roles: a “profit-maximizing ‘businessperson‴ and a "nonstrategic ‘friend’.” Players take up the role of the businessperson initially. If cooperation continues sufficiently in the trial phase, the players switch their role to “friend” and engage in unconditional cooperation, meaning that they always cooperate despite their “defection.”

Montgomery’s Embeddedness model sheds light on the previously neglected aspect of trust. However, it is not infallible. First, the model is vulnerable to situations where malicious players pretend to be friends and exploit good intentions after friendship is established. Even if we commit ourselves to a relationship, threats of sanction should be guaranteed to some extent.

Further, one can consider the “mirror image” of Montgomery’s model. In this case, players generously accept newcomers even if the newcomers appear to have dubious intentions or competence. Here, trustors then base selection of partners on repeated interactions. For example, “trial basis employment” a system where recruits who perform well can obtain more permanent employment once they have successfully completed a trial period, is an extension of this partner selection strategy. During the trial period, however, employers are allowed to dismiss any recruits whose character or behavior is problematic and can do so without complicated procedure.

In sum, the balance between forgiveness and punishment is crucial for generous trust relationships. This balance is partially demonstrated in Montgomery’s two-stage model, but the “mirror image” of the model should be supplemented.

We should distinguish two different processes where generosity works: the matching process and the commitment (or exit) process. In the matching process, people may be generous when accepting an unknown person as a new partner; they are optimistic about the potential a new partner brings. We call this matching generosity (corresponding to the “mirror image” of Montgomery’s model). On the other hand, people may become tolerant once they recognize a partner as friend. In this case, the other party is more forgiving of a partner’s mistake and less motivated to leave or exit the relationship. We will refer to this as exit generosity (corresponding to the original Montgomery’s model).

Here, we integrate these two patterns of trust behavior into a coherent theoretical model. By using Montgomery's model, we investigate the types of trust strategies that thrive in the face of uncertainties about others’ intentions and competence, and, under what conditions.

## Previous Works

### Trust

One central theory in trust studies is the Emancipation Theory of Trust, proposed by Yamagishi [3]. He argues that, the trustors who tend to exhibit a high level of trust apt to apply a strict criteria in choosing their potential partners; they tend to break off their relationship immediately when the partner turns out to be a betrayer and seek alternatives. In brief, a high level of trust “emancipates” people from committed relationships. Their strategy is comprised of two parts: partner selection and committed relationship. Trustors select new partners more stringently and leave committed relationships when a partner betrays them or makes a mistake. This principle of trust has been analyzed by agent-based simulation using the out-for-tat (OFT) strategy—a strategy found to be effective at maintaining cooperation [7, 8]. The other line of trust studies analyzes trust relationship by a *trust game*, which is an asymmetric and non-simultaneous sequential game created by Berg et al. to measure the level of trust between game participants [4, 9–12]. Previous work on the trust game have clarified the importance of reputation and punishment to develop trust [13–16].

Both types of trust studies rely on the severity of a partner’s failures during the trustor's selection process and whether or not the trustor terminates the committed relationship. However, these trust studies focus primarily on a specific aspect of trust: trust in intention. When deciding to trust others, we are confronted with two types of uncertainties: one is trustees’ intention; the other is is a trustee's competence, defined here as the possibility of accomplishing a given task. Failure to maintain trust on behalf of the trustee can be caused by an intentional betrayal or an accidental failure; our proposed alternative viable strategy will take these elements into account.

### Uncertainty in trust games

Game theory and simulation studies have also pointed out the risk of uncertainties and the importance of generosity in uncertain situations. Uncertainties have been introduced into games as noise, which occurs exogenously with a given (usually slight) probability [17]. In the games with noise, generous tit-for-tat (GTFT) strategy and Forgiver strategy are found to outperform the original tit-for-tat (TFT) strategy, which is known to be one of the most effective strategies in games without noise [18–23]. Once uncertainties (failure or mistakes) are introduced, original TFTs go to long-run mutual backbiting. Thus, the close relationship and reputation mechanisms cease to work by just a small noise. In contrast, GTFT punishes a defector based on a certain probability. The Forgiver retaliates quickly after the opponent has defected and stops retaliation immediately, irrespective of the opponent’s move right after he/she defected. Thus these two generous strategies can return to mutual cooperation soon after a mistake has been committed in different ways.

Although studies on games with noise incorporate the idea of uncertainty, they do not examine trust as a socially embedded relationship. Most of them focus on iterated two-person prisoners’ dilemma games and random matching. Our model integrates these two streams into one coherent model. Different from the emancipation theory of trust, we examine trust in highly uncertain environment. In contrast to the dyadic game theory, we examine socially embedded trust relationships, that is, in the context of partner selection and the commitment process.

This framework leads to several questions. The first question relates to the amount of noise. Note that competence here is somewhat different from noise. In previous studies, noise is assumed to be a small perturbation. In contrast, competence is not a small perturbation as we cannot imagine people who always perform perfectly. However, some studies report that the fitness of players adopting GTFT decreases as the amount of noise increases [19, 24, 25]. Therefore, the remaining conundrum is as follows: Under what condition is it rational for a trustor to be generous despite a trustee’s failure? How much uncertainty can generous trust tolerate?

The second question relates to the relationship between generosity and social embeddedness or specifically an option to opt out. Studies on a dyadic game with noisy situations suggest that generosity is needed to some extent. In contrast, emancipation theory of trust claims that one should opt out of the relationship with the defector if provided with an option for selecting a new partner. What remains unclear is whether it is still best to opt out even if there is noise in the sense of the possibility that a partner fails by accident, not by intention. Or conversely, is it still rational to be tolerant of partner's failure when one has an option to opt out? Furthermore, even if there is a room for generosity, what type of generosity is essential? Generosity in the matching process or that in the commitment process or both?

To tackle these questions, we analyze trust game with uncertainties of competence and the partner-selecting process (matching and commitment process).

## Materials and Methods

### Outline of the two models

In this section, we outline the idea behind our models. We adopt a two-stage analytic strategy. The final goal is to analyze the model with a “full” setting. The full model, however, is too complicated to be mathematically tractable. Therefore, it is useful to analyze a simplified model to obtain a clear understanding of the mechanism before moving to the full model.

In our full model (Model 2), we analyze a social system with N players. Players encounter each other at random and enter into trust relationships as long as the trustor trusts the recipient in this matching stage. Trust relationship is also monitored by the trustor and can be resolved. Thus, there are two main processes in the model, the matching process and the commitment or exit process. Correspondingly, we can differentiate generosity in the matching process and that in the exit process. Hereafter, we will call these matching generosity and exit generosity, respectively.

### Model 1

We are interested in the balance between punishment and forgiveness (generally) and matching generosity and exit generosity (specifically). For the former purpose, we simplify the model (Model 1) by reducing it to the dyadic trust relationship and discarding the matching process. After obtaining a firm understanding of the balance between punishment and forgiveness in the commitment process, we delve into the full model with the matching and commitment processes.

**Fig 1:**
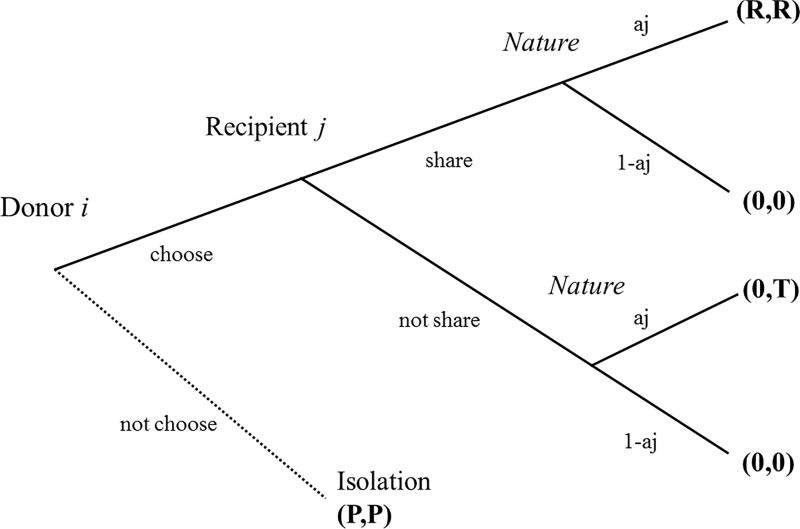
Game-tree of modified trust game.

In Model 1, a dyadic, two-player trust relationship is modeled by a repeated “modified trust game” (see Fig 1). A sub-game incorporates the idea of probabilistic success and information asymmetry, whereas the entire game comprising repeated sub-games enables the modeling of forgiveness and generous trust.

The entire game comprises100 sub-game rounds, in each of which the donor makes the first move to decide whether to entrust his or her resource to recipients, as in the usual trust game. (It is easy to extend this infinitely by introducing a time discount. We do not this for two reasons: first, for simplicity; second, it is more consistent with Model 2.)

The sub-game proceeds in the following way: if the donors decide not to trust, the game ends and the two parties end up with the same “isolation” payoff P. If the donors decide to trust, the recipients take a turn and decide whether to honor the trust. After their decision, “nature” takes a turn, representing the idea of probabilistic success. Recipients, regardless of their decision whether to honor the trust, attempt to increase the resource at hand; however, this attempt fails with a certain probability 1 − *a*_*j*_. Hence, *a*_*j*_ can be interpreted as recipients’ competence. If recipients decide to honor the trust and succeed in increasing resources, the augmented resource is divided equally between the two parties, leading to payoff R. If recipients decide to defect and succeed in their businesses, they monopolize the benefit to obtain payoff T, while donors gain no payoff. If recipients fail to succeed, the two parties end up with no payoff, whether the recipients make up their mind in advance to honor the trust. All these processes are shown in Fig 1. The payoffs satisfy the conditions T > R > P > 0 and 2R > T. Here, we assign values such that P = 1; R = 4; and T = 6. Therefore, mutual cooperation is better than mutual defection in both roles, but a donor’s distrust following a recipient’s defect is the best action in a single round.

The sub-game payoff is summaraized in the below matrix.

$$\begin{array}{l} \;share\;not\;share\\ choose\;(a_{j}R,a_{j}R)\;(0,a_{j}T)\\ not\;choose\;(P,P)\;(P,P) \end{array}$$

The structure of the entire game is described in the following. After one sub-game round ends, donors decide whether to continue to invest the recipients or not in light of the recipients’ previous behaviors recorded in the “history.” Here, information asymmetry is assumed. That is, donors cannot discriminate between the cases where recipients intentionally betrayed and those where recipients had a good intention to cooperate but failed for accidental reasons as donors can know only observable results—i.e., how much recipients return to them. Accordingly, recipients have a good reputation (history *C*) only if they intend to cooperate and succeed. Otherwise, since recipients return nothing to donors, their history becomes defection (*D*). For simplicity, we assume that the history records only one previous behavior.

If donors decide to continue their relationship, the two parties play the same sub-game and gain each payoff as the game results. In contrast, if donors decide not to invest, the relationship ends and is never re-established. The relationship may be also dissolved for some exogenous reasons with a certain probability (*d* = 0.1). In either case, donors and recipients receive the same payoff P over the remaining rounds.

We restrict the donor’s strategies to three in order to make the model tractable and simultaneously give it minimal complexity that enables us to examine the balance between punishment and generosity. The three strategies are called the high, middle, and low generosity strategies (*H*, *M*, and *L*, respectively). If the donor selects *H*, they always choose *C* regardless of the recipient history. If *M* is selected, the donor can forgive only one defection and an exit is prescribed if the recipient fails two times in consecutive rounds. If *L* is adopted, the donor exits immediately after a single recipient failure. Recipients can only decide whether to cooperate (*C* or *D*).

The entire game payoffs is summarized in the matrix form. The concrete payoff value in each cell can be calculated using the theory of finite Markov chain (S1 Fig). Furthermore, the probability that Player 1 chooses *H* or *M* is denoted by *p*_1_ and *p*_2_, respectively. Likewise, *C* and *D* for Player 2 (recipient)’s strategy means cooperation and defect. *q* represents the probability that player 2 chooses *C*.

$$\begin{array}{l} \;C\;D\\ H\;(\alpha_{1},\;\alpha_{2})\;(b_{1},\;b_{2})\\ M\;(c_{1},\;c_{2})\;(d_{1},\;d_{2})\\ L\;(e_{1},\;e_{2})\;(f_{1},\;f_{2}) \end{array}$$

Using these notations, we describe the condition under which *H* or/and *M* is viable. In other words, we want to show that there is a mixed Nash equilibrium in which Player 1 takes mixed strategy of *H and M* and Player 2 uses *C and D*; In this case, we can say Player 1’s strategy more or less exhibits forgiveness.

The conditions for the mixed Nash equilibrium in the modified trust game are as follows:

$$0\;<\frac{d_{1}-b_{1}}{a_{1}-c_{1}+d_{1}-b_{1}}<1$$ (C 1)

$$\frac{{(d}_{1}-b_{1)}(a_{1}-e_{1})+(a_{1}-c_{1})(b_{1}-f_{1})}{a_{1}-c_{1}+d_{1}-b_{1}}>0$$ (C 2)

$$0<\frac{\left(c_{2}-d_{2}\right)}{\left(c_{2}-d_{2})+(b_{2}-a_{2}\right)}<1$$ (C 3)

The proof is shown in S1 Fig.

Using these inequalities and the result of calculating the concrete payoff values, several propositions can be derived (the derivation is detailed in S1 Fig).

1. (C 2) is satisfied when *a* > 0.25(this also makes (C 1) satisfied). This is intuitively obivious because the cooperation does not make sense unless *aR* > *P*(4*a* > 1). In addition to that, Note that the state (*H*, *C*) is Pareto efficient when *a* > 0.25, but that it never constitutes a pure Nash equilibrium. This is because given a donor’s choice of *H*, *D* always yields a larger payoff to the recipient. This is the dilemma aspect of the modified trust game.

2. (C 3) is satisfied when *a* > 0.47. We denote this value a threshold for cooperation *λ* regarding competence level *a*. The possibility of cooperation does not emerge until *a* is beyond *λ*. This is understandable. When *a* > *λ*, *c*_2_ is larger than *d*_2_(see S1 Fig). Thus, the recipient, given a donor’s choice of *M*, would choose *C* (the possibility of cooperation and generosity).

3. For the mixed Nash equilibrium, as competence level *a* increases, the probability of cooperation increases, as does the probability of implementing the high generosity strategy rather than the middle generosity strategy. When *a* > 0.54, the mixed Nash equilibrium is the only one equilibrium in this system.

The above results are summarized in Table 1.

**Table 1:** Ranges of Nash equilibrium and Pareto efficiency derived from the mathematical analysis.

	*a* < 0.26	0.26 ≤ *a* < 0.47	0.47 ≤ *a* < 0.54	0.54 < *a*
*Nash Equilibria*	*(L*, *D)* is only Nash equilibrium.	*(L*, *D)* is only Nash equilibrium.	*(L*, *D)* and the mixed strategy (*H* and *M*; *C* and *D*) constitute Nash equilibria.	(*H* and *M*; *C* and *D*) constitute Nash equilibria. The ratio of *H* increases as *a* increases.
*(H*,*C) is Pareto efficient*	*no*	*yes*	*yes*	*yes*

In sum, the mathematical analysis yields some basic insights into the mechanism of *generous trust*. First, the uncertain conditions make generous trust a viable option. However, if social uncertainties are too vast, the possibility of generous trust is destroyed even though the combination of high generosity and cooperation is desirable for both parties under certain conditions. Second, the high generosity strategy is viable only when co-existing with the middle generosity strategy, which uses a moderate means of punishment, implying a balance between generosity and punishment is vital.

### Model 2

However, because the mathematical analysis is too simplified, it leaves several interesting questions unclear. Is generous trust still viable even if a donor can freely choose a new partner? If so, what role differentiations emerge among matching generosity, exit generosity, and super generosity, which are analogous to HG in Model 1? These questions are addressed by Model 2.

This section illustrates the agent-based simulation and its results. Agent-based simulation is a kind of numerical simulation in the field of social science. It enables us to analyze the dynamic process of social realities or social phenomena including human interactions that many social scientists focus on. We can represent people as “agents” in the models that have heterogeneity of preferences or characteristics (Gilbert [26]). We construct the agent-based simulation with C++ (simulation codes available from the authors). We add the matching process to the above mathematical model. In addition, we reformulate strategies involving matching generosity and exit generosity, thereby enabling us to examine the balance between them.

**Fig 2:**
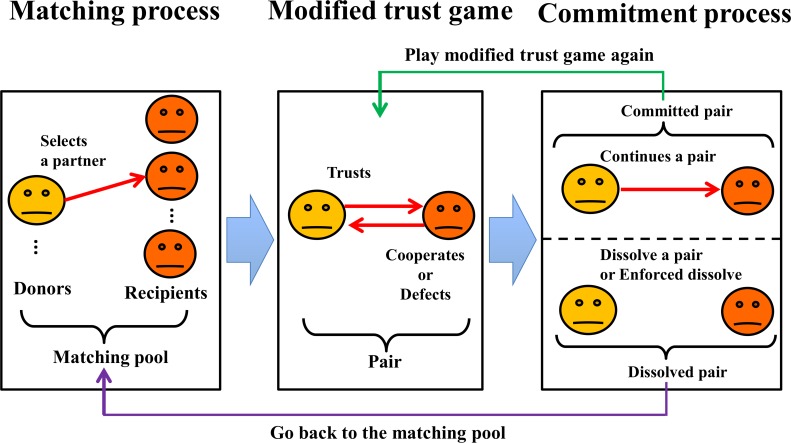
Procedure of the agent-based simulation.

Fig 2 shows the process of one round. At the beginning of the round, each donor chooses a partner in a matching pool according to his or her matching generosity. We call this phase matching process. After the paired players play the modified trust game explained above, the donor decides whether to continue or dissolve the relationship according to his or her exit generosity. We call this phase commitment (or exit) process. If the donor decides to continue the relationship he or she plays the game with the current partner again. If not or if the relationship is dissolved exogenously (a certain probability *d*), both players go back to the matching pool. The donor has to choose a new partner while the recipient has to wait to be selected as a partner at the beginning of the next round. After this process repeats *L* rounds, the roles of both players evolve.

In the matching and commitment processes, donors select partners according to their own “generous strategies”. These are differentiated by each distinct process, the matching process or the commitment process. Note that donors’ memory (history) is restricted to only the last rounds in this agent-based simulation. In both processes, the donors refer to potential (current) partners’ histories from only the last round. The four strategies for donors are as follows: (1) Strict Trust strategy (**ST**); a donor chooses only a partner with good previous results and maintains the partnership only when the recipient reciprocates. (2) Super Generous strategy (**SG**); a donor chooses a partner regardless of his or her previous result and continues the relationship even with the partner who has defected. The other two strategies are related to *generous trust*. (3) Matching Generous strategy (**MG**) is a generous strategy in the matching process. A donor chooses a partner in which to form a relationship even if he or she has failed in the previous round. However, this donor is strict in the commitment process to dissolve their relationship soon after the recipient fails in honoring the trust. (4) Exit Generous strategy (**EG**) is a generous strategy in the commitment process. A donor is generous in regards to a partner’s defection and remains in the relationship, although this donor is strict in the matching process (see Table 2). On the other hand, the recipients have three types of intention (*x*_*i*_ ∈ {0, 0.5, 1}, which represent the probabilities of honoring the trust). Thus, the donors’ strategies are classified into four types and the recipients’ strategies into three types. In addition, we assume that all players have the same competence for simplicity. This simplification clarifies what is happening in the dynamics of strategies. We need further research on the game with complex strategies and different levels of competence.

**Table 2:** Correspondence between a recipient’s partner-choice criterion and strategies.

		Commitment Process	
	History	Only C	C or D
Matching Process	Only C	Strict Trust (ST)	Exit Generous (EG)
	C or D	Matching Generous (MG)	Super Generous (SG)

After every *L* rounds, the distribution changes by imitation dynamics wherein the players imitate the more successful strategy with a certain mutation error (*M*). Thus, the donor’s generosity and recipient’s intention can change. However, the recipient’s competence does not change because it is assumed as a natural endowment.

We continuously set payoff values as P = 1, *R* = 4, *T* = 6, and *B* = 0.1. In addition, we set a mutation rate of *M* = 0.025 and rounds per evolution at *L* = 100. We have choosen this mutation rate level for the following reason. First of all, the qualitative results do not change at the sufficient low level of the mutation rate (up to about 0.18), so we have choosen the fairly small rate as common practice do. Although the results become slightly different beyond 0.18, too high mutation rate is rather highly unrealistic in evolutionary setting and therefore unacceptable. Moreover, we have observed that the convergences occur with regard to the main processes such as what kinds of pair is matched, who is isolated etc. (see simulation parts) around 50th round. On the safe side we have choosen the number of round to 100.

The benchmark case is 250 donors and 250 recipients. At the very beginning of the game, players are randomly assigned their strategies ($g_{i}^{M}$, $g_{i}^{E}$, *x*_*i*_) according to a uniform distribution. We find the following two main results. Note that these results match to the findings from the mathematical analysis of Model 1.

1. A threshold of competence exists and needs to exceed 0.60 to sustain trust and generosity.
2. When players build trusting relationships, the EG and SG strategies coevolve.

**Fig 3:**
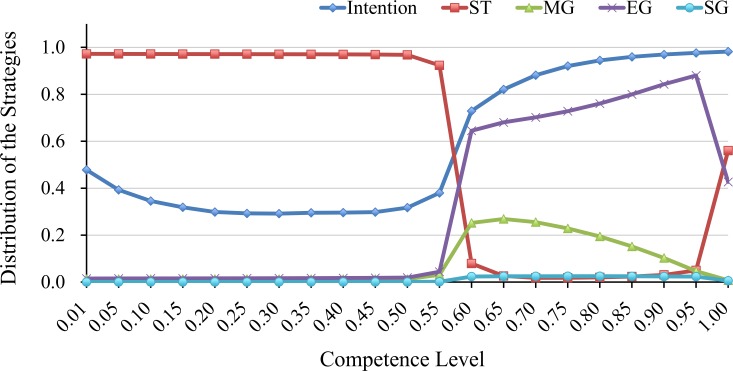
The relationship between competence and intentions. The X-axis is each competence level. The Y-axis is the distribution of the strategies. We conducted 100 trials (each trial has 100 evolutions) at each competence value. The points are the averages of the trials.

The remaining parts explain these main results. First, we run the simulation analysis with different levels of competence to examine the relationship between the level of uncertainty and the type of trust. Fig 3 shows that the levels of intention to cooperation and proportions of each strategies at different levels of competence. As Fig 3 reveals, the threshold needed to sustain cooperation falls within the range between 0.55–0.60. Simultaneously, when cooperation is maintained, generosity and trust are not corrupt. Finally, a brief comment should be added on the result when *a* > 0.95. The result that ST and EG strategy coexist in this range corresponds to that of previous works. OFT strategy is somewhat parallel to ST strategy and Fogiver and GTFT is to EG strategy. The previous works show that OFT, Forgiver and GTFT flourish in the range of just small noise. In the situation which unintentional mistakes barely happen, strictness or exit generosity is required to maintain cooperation because most of the failure should be interpreted as malicious. However, this is only the case in a rather small range of competence value *a*. Otherwise, two types of generous trust strategies can coexist as our result shows.

**Fig 4:**
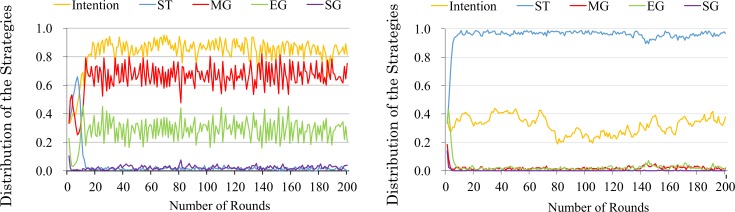
The distribution of trust strategies. The X-axis is each competence level. The Y-axis is the distribution of the strategies. The right and left graphs represent the typical dynamics at *a*^*m*^ = 0.65 and *a*^*m*^ = 0.55, respectively.

**Fig 5:**
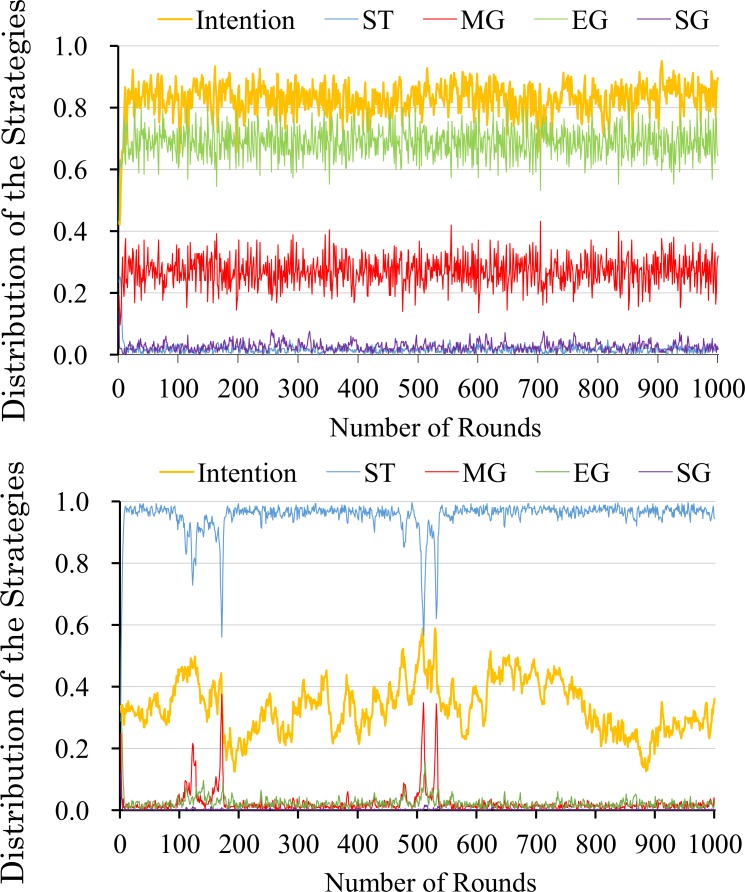
The distribution of strategies. The X-axis is the generations. The Y-axis is the distribution of the strategies.

Further, to clarify which strategy flourishes on the condition that a sufficient number of recipients have a good intention to cooperate, we examine the distribution of strategies. Fig 4 shows a typical case of the results. Note that we calculate the standard deviations of both competence values to check the stability among 100 trials. When the competence value is 0.55, the standard deviation of intention is 0.0479, ST is 0.1272, MG is 0.0263, EG is 0.0977, and SG is 0.0035. When the competence value is 0.65, the standard deviation of intention is 0.0113, ST is 0.0204, MG is 0.0031, EG is 0.0170, and SG is 0.0009. Moreover, the order of the proportions of the strategies do not change between trials. From these results, it can be said to be stable among trials. Although Fig 4 represents the result of up to 200 rounds, the subsequent dynamics are stable (see Fig 5). Because of a stochastic factor (mutation rate) in the evolution process, the dynamic became temporarily unstable. For example in the evolution process, recipients with good intention rapidly increase by mutation (i.e. at 150^th^ generation and at 500^th^ generation in the above graph of Fig 5), and ST strategy tends to decrease. However this instability is not enough to change the overall distribution of strategies. In the situation where recipients are sufficiently cooperative (competence greater than 0.65), the EG and SG strategies coevolve. In contrast, when, competence is 0.55 (and less than 0.55), the ST strategy, which is extremely strict on others’ defects in the matching and commitment processes, is dominant in the population.

### Discussion of the simulation

**Fig 6:**
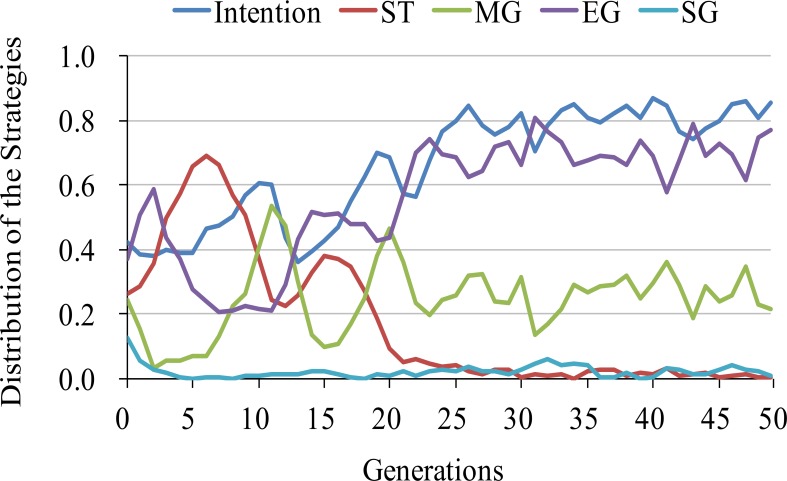
The distribution of strategies in the initial phases. This graph shows a typical trial with the value at *a*^*m*^ = 0.65. The X-axis is the generations. The Y-axis is the distribution of the strategies.

We discuss the mechanism of the coexistence of the MG and EG strategies. The dynamics are divided into three parts: strict selection, a rise in matching generosity, and a stability of generosity in either process. These phases correspond to the 1^st^–5^th^ generations, 6^th^–15^th^ generations, and 16^th^ generations, respectively (see Fig 6).

**Fig 7:**
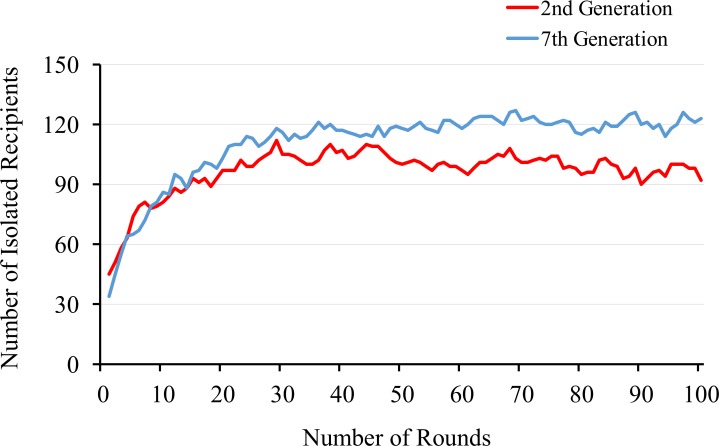
The numbers of the isolated recipients with bad history and good intention. The X-axis represents rounds and the Y-axis represents the number of isolated recipients.

In the first phase, because recipients’ intention is not so high yet (0.45–0.55), which is close to the initial distribution, defects are more likely to occur from malice than from incompetence. In this situation, it is better for the donors to leave a partner who has defected and stay alone, rather than associate with players who have bad histories. Thus, strict selection is needed to not be exploited and ST strategy is increasing. This leads the recipients to increase the levels of intention. In the second phase there are many recipients with good intentions. The ST strategy abandons partnerships in the face of failures, including those that are simple mistakes. In the matching pool, on an average, 120 out of 190 recipients (see Fig 7) have good intentions and a bad history. The ST strategy never picks up such recipients. On the other hand in this situation, MG strategy effectively makes use of such recipients who have good intentions, but also have a bad history. This strategy picks such a partner from the matching pool. In the situation where recipients’ intention to cooperate increases, defections are more likely to come from incompetence than from malice. Thus, in this situation, donors could receive returns in next round with a probability rate of 0.6. Therefore, the donors prefer having a partner to staying alone.

In the third phase, most of the recipients have good intention. Thus keeping their relationships is more profitable than being isolated. MG strategy is always paired. Even if they abandon a partner, they are guaranteed of finding a new partner in the next round unlike ST strategy. Similarly EG strategy keeps the relationships with the same partner for a long period. In contrast, EG strategy have a risk of being isolated because if their relationship is broken by exogenous factors, it is difficult to find a new good partner. Thus, the MG strategy has a slight advantage over the EG strategy. However, a decrease in EG strategy use leads to a corresponding decrease in recipients’ intention. Therefore, the EG strategy, MG strategy and recipient’s cooperation strike a balance. Because previous works introduce only small noise such as competence, which is nearly equal to one in Fig 3, they do not recognize this combination of error-correcting strategies.

This result is obtained from a trial at *a*^*m*^ = 0.65. This figure represents the numbers of the isolated recipients with bad history and good intention in the second generation (an example of the first phase) and in the seventh generation (an example of the second phase). This graph illustrates that the number of isolated trustees with good intention and bad history in the second phase is larger than that of in the first phase.

## Conclusion

In summary, our mathematical analysis and simulation studies illustrate that there exists a threshold for cooperation and generosity. Two implications can be seen. First, the fact that the threshold is above the minimum level for the existence of a Pareto-efficient social state implies that social uncertainties inevitably cause inefficiency to a certain extent. Admitting this, society can still tolerate a considerable amount of uncertainty with the condition that people adopt generous strategies. Furthermore, the mathematical analysis shows that while the strict strategy (low generosity) fails when there are greater and/or more social uncertainties, a high generosity strategy also cannot stand alone because it lacks any measures to punish betrayers, suggesting that a well-functioning society requires an optimal mix of generosity and punishment.

Further, our simulation study suggests the importance of “functionally differentiated trust,” that is, the distinction between matching generosity and exiting generosity. Let us illustrate this idea in comparison with Montgomery’s work. His model can be interpreted as formulating exiting generosity in our model in the sense that the trustor forgives the partner’s “defection” after selecting the partner as trustworthy in the matching stage. However, the risk of being exploited never vanishes in our model. In addition, a very strict selection in the matching process can make it difficult to find a partner. This strict selection in exit generosity also leads potential trustees who have failed by accident to become isolated. Here, trustors with matching generosity take a leap of faith in establishing relationships with such trustees and give them a chance. The coexistence of exiting and matching generous strategies can achieve a balance between generosity and punishment, thus leading to a well functioning society.

## Supporting Information

S1 Fig The Markov chain of payoffs. Each node represents a state. *IN*: the initial state. *RR*: means *R* was achieved in the last consecutive two rounds (or *R* achieved in the first round). *RF*: *R* was achieved in the last two rounds, but failure occurred in the last round. *FF*: *F* occurred in the last consecutive two rounds. *P*: the two players broke up and no longer interact. Each edge denotes a path with a probability such as *a*, *da*, and so on.
(DOCX)

## References

[1] Frank RH. Passions within Reason: The Strategic Role of the Emotions. W. W. Norton & Company; 1988.

[2] Bachmann R, Zaheer A, eds. Handbook of Trust Research. Edward Elger Publishing; 2006 pp. 165–186.

[3] Yamagishi T. Trust The Evolutionary Game of Mind and Society. New York: Springer; 2011.

[4] Berg J, Dickhaut J, McCabe K. Trust, Reciprocity, and Social History. Games. Econ. Behav. 1995; 10(1): 122–142.

[5] Uzzi B. The Sources and Consequences of Embeddedness for the Economic Performance of Organizations: The Network Effect. Am. Sociol. Rev. 1992; 61(4): 674–698.

[6] Montgomery JD. Toward a Role-Theoretic Conception of Embeddedness. Am. J. Sociol. 1998; 104(1): 92–125.

[7] Hayashi N. From TIT-for-TAT to OUT-for-TAT (in Japanese). Sociol. Theor. Method. 1993; 8(1): 19–32.

[8] Hayashi N, Yamagishi T. Selective Play: Choosing Partners in an Uncertain World. Pers. Soc. Psychol. Rev. 1998; 2(4): 276–289. 10.1207/s15327957pspr0204_4 15647134

[9] Dasgupta P. Trust as a Commodity In: Gambetta D, ed. Trust: Making and Breaking Cooperative Relations, electronic edition. Department of Sociology, University of Oxford; 2000 pp. 49–72.

[10] Coleman JS. Foundations of Social Theory. Harvard: Harvard University Press; 1990.

[11] McCabe KA, Smith VL, LePore M. Intentionality Detection and “Mindreading”: Why Does Game form Matter? Proc. Natl. Acad. Sci. 2000; 97(8): 4404–4409. 10760306 10.1073/pnas.97.8.4404 PMC18254

[12] Gambetta D. Can We Trust Trust? In: Gambetta D, ed. Trust: Making and Breaking Cooperative Relations, electronic edition. Department of Sociology, University of Oxford; 2000 pp. 213–237.

[13] Lahno B. Trust and Strategic Rationality. Ration. and Soc. 1995; 7(4): 442–464.

[14] Buskens V. The Social Structure of Trust. Soc. Networks. 1998; 20(3): 265–289.

[15] Bravo G, Tamburino L. The Evolution of Trust in Non-Simultaneous Exchange Situations. Ration. and Soc. 2008; 20(1): 85–113.

[16] Matsuda M, Yamagishi T. Trust and Cooperation: An Experimental Study of PD with Choice of Dependence (in Japanese). Jpn. Psychol. Res. 2001; 72 (5): 413–421. 10.4992/jjpsy.72.413 11883329

[17] Axelrod R. Launching “The Evolution of Cooperation”. J. Theor. Biol. 2012; 299: 21–24. 10.1016/j.jtbi.2011.04.015 21540040

[18] Molander P. The Optional Level of Generosity in a Selfish, Uncertain Environment. J. Confl. Resolut. 1985; 29(4): 611–618.

[19] Nowak M, Sigmund K. Tit-for-Tat in Heterogeneous Populations. Nature.1992; 355(6357): 250–253.

[20] Matsushima H. Interlinkage and Generous Tit-For-Tat Strategy. Jpn. Econ. Rev. 2014; 65(1): 116–121.

[21] Wu J, Axelrod R. How to Cope with Noise in the Iterated Prisoner’s Dilemma. J. Confl. Resolut. 1985; 39(1): 183–189.

[22] Boyd R. Mistakes Allow Evolutionary Stability in the Repeated Prisoner’s Dilemma Game. J. Theor. Biol. 1989; 136(1): 47–56. 2779259 10.1016/s0022-5193(89)80188-2

[23] Zagorsky BM, Reiter JG, Chatterjee K, Nowak M. Forgiver Triumphs in Alternating Prisoner’s Dilemma. PLoS ONE. 2013; 8(12): e80814 10.1371/journal.pone.0080814 24349017 PMC3861238

[24] Axelrod R, Dion D. The Further Evolution of Cooperation. Science. 1998; 242(4884): 1385–1390. 10.1126/science.242.4884.1385 17802133

[25] Nowak M, Sigmund K. A Strategy of Win-Stay, Lose-Shift that Outperforms Tit-for-Tat in the Prisoner’s Dilemma Game. Nature. 1993; 364(6432): 56–58. 10.1038/364056a0 8316296

[26] Gilbert N. Agent-Based Models. SAGE Publications; 2008.

