# Correction: The Condition for Generous Trust

**DOI:** 10.1371/journal.pone.0172597

**Published:** 2017-02-15

**Authors:** Shinya Obayashi, Yusuke Inagaki, Hiroki Takikawa

All three authors’ names are listed incorrectly. The correct names are: Shinya Obayashi, Yusuke Inagaki, Hiroki Takikawa. The correct citation is: Obayashi S, Inagaki Y, Takikawa H (2016) The Condition for Generous Trust. PLoS ONE 11(11): e0166437. doi:10.1371/journal.pone.0166437
