# Supplementary material for: The Condition for Generous Trust
Source: PLoS One. 2016 Nov 28;11(11):e0166437. doi: 10.1371/journal.pone.0166437 (PMC5125584; doi:10.1371/journal.pone.0166437)
Supplement: S1 Fig — Each node represents a state. IN: the initial state. RR: means R was achieved in the last consecutive two rounds (or R achieved in the first round). RF: R was achieved in the last two rounds, but failure occurred in the last round. FF: F occurred in the last consecutive two rounds. P: the two players broke up and no longer interact. Each edge denotes a path with a probability such as a, da, and so on. (DOCX) [file pone.0166437.s001.docx]

Supplementary Information 1. Mathematical Analysis

*The condition for mixed Nash equilibrium*

　Let us examine the conditions for Player 1’ s strategy to be a mixed strategy of $H and M$.

　First of all, Player 1’s expected payoff is as follows:

$p_{1}\left[ qa_{1}+\left( 1-q \right)b_{1}-qe_{1}-\left( 1-q \right)f_{1} \right]+p_{2}[qc_{1}+\left( 1-q \right)d_{1}-qe_{1}+\left( 1-q \right)f_{1}]$+$[qe_{1}+\left( 1-q \right)f_{1}]$ (S 1)

The conditions for Player 1’ s strategy to be a mixed strategy of $H and M$ are two-fold.

1) $q^{*}$ falls in the interval$(0, 1)$ such that the coefficients of $p1$ and $p2$ in (S 1) are equal:

$q^{*}a_{1}+\left( 1-q^{*} \right)b_{1}=q^{*}c_{1}+\left( 1-q^{*} \right)d_{1}\Leftrightarrow q^{*}=\frac{d_{1}-b_{1}}{a_{1}-c_{1}+d_{1}-b_{1}}$; hence, 0 <$\frac{d_{1}-b_{1}}{a_{1}-c_{1}+d_{1}-b_{1}}<1$

This inequalities are the condition (C 1).

2) The expected payoff of H is greater than of L on the condition that Player 2 takes $q^{*}$:

$$q^{*}\left( a_{1}-e_{1} \right)+\left( 1-q^{*} \right)\left( b_{1}-f_{1} \right)>0$$

Using $q^{*}=\frac{d_{1}-b_{1}}{a_{1}-c_{1}+d_{1}-b_{1}}$, the condition (C 2 ) is obtained:

$\frac{{(d}_{1}-b_{1)}\left( a_{1}-e_{1} \right)+(a_{1}-c_{1})(b_{1}-f_{1})}{a_{1}-c_{1}+d_{1}-b_{1}}>0$ (C 2)

Next, let’s see the conditions for player2’s strategy to be mixed one.

Player 2’s expected payoff is as follows:

$q\{p_{1}\left[ \left( a_{2}-e_{2} \right)+\left( f_{2}-b_{2} \right)]+p_{2}\left[ \left( c_{2}-e_{2} \right)+\left( f_{2}-d_{2} \right) \right]+e_{2}-f_{2} \right\}+{[p}_{1}b_{2}+p_{2}d_{2}+\left( 1-p_{1}-p_{2} \right)f_{2}]$ (S 2)

The conditions for Player 2’ s strategy to be the mixed strategy of $C and D$ is that the coefficient of $q$ is zero:

$p_{1}\left[ (a_{2}-e_{2} \right)+\left( f_{2}-b_{2} \right)]+p_{2}\left[ \left( c_{2}-e_{2} \right)+\left( f_{2}-d_{2} \right) \right]+e_{2}-f_{2}=0$ (S 3)

Furthermore, for Player 1’s strategy to be the mixed strategy of $H and M$ in this case, there exist $p_{1}^{*} and p_{2}^{*}$ such that $p_{1}^{*}+ p_{2}^{*}=1 and 0<p_{1}^{*}, p_{2}^{*}<1$. Combining this with (S 3), we obtain

$$p_{1}^{*}=\frac{\left( c_{2}-d_{2} \right)}{\left( c_{2}-d_{2} \right)+\left( a_{2}-b_{2} \right)}$$

Hence, the condition (C 3) is:

$0<\frac{\left( c_{2}-d_{2} \right)}{\left( c_{2}-d_{2} \right)+\left( a_{2}-b_{2} \right)}<1$ (C 3)

*Calculations of the payoffs*

Each payoff in the matrix can be calculated using the theory of finite Markov chain. This can be illustrated by focusing on the calculation of $c_{2}$ (the payoff for Player 2 in the state $(M, C)$).

The stochastic process evoked by the mutual move M and C can be modeled by absorbing the Markov chain in Fig S 1. This also can be represented by a stochastic matrix (Fig S 2). Using this matrix, we calculate the fundamental matrix $M,$ and the first row of $M$ gives the expected number of visits to each state from the state $IN$. This is the vector $(1,\frac{a+ad-a^{2}d}{1-ad-ad^{2}+a^{2}d^{2}},\frac{1-a}{1 d-ad^{2} +a^{2}d^{2}},\frac{d-2ad+a^{2}d}{1-ad-ad^{2}+a^{2}d^{2}})$, whose elements represent the expected number of visits to$IN, RR, RF,\mathrm{and} FF$, respectively (remember $d$ denotes the probability of breaking up). Using this, we can calculate the payoff for Player 2; $c_{2}=\frac{a+ad-a^{2}d}{1-ad-ad^{2}+a^{2}d^{2}}R+\frac{1-a}{1 d-ad^{2} +a^{2}d^{2}}F+\frac{d-2ad+a^{2}d}{1-ad-ad^{2}+a^{2}d^{2}}F+\left( 100-\frac{a+ad-a^{2}d}{1-ad-ad^{2}+a^{2}d^{2}}-\frac{1-a}{1 d-ad^{2} +a^{2}d^{2}}-\frac{d-2ad+a^{2}d}{1-ad-ad^{2}+a^{2}d^{2}} \right)P.$ Note the expected number of visits to P is 100 − the expected number of visits to other states. All the payoffs are calculated in the same way.

**S 1 Fig. The Markov chain of payoffs.** Each node represents a state. $IN$: the initial state. $RR$: means $R$ was achieved in the last consecutive two rounds (or $R$ achieved in the first round). $RF$: $R$was achieved in the last two rounds, but failure occurred in the last round. $FF$: $F$occurred in the last consecutive two rounds. $P$: the two players broke up and no longer interact. Each edge denotes a path with a probability such as $a, da,$ and so on.

*The derivations of the propositions*

Now, we can derive the proposition described in the main text.

1. With regards to (C 2), by inserting calculated values into (C 2), we obtain $0<\frac{a\left( -13.85+75.82a-88.2a^{2}+26.24a^{3} \right)}{1-2.61a+2.35a^{2}-0.73a^{3}}$. This inequality holds when $a>0.25$. It is also obvious that $a_{1}>c_{1}>e_{1} and b_{1}<d_{1}<f_{1}$ on the condition that $a>0.25$, making (C1) satisfied.

2. $c_{2}-d_{2}=$ $(a(-6.15+17.43a-9.23a^{2}))/(1-1.71a+0.81a^{2})$. If $a>0.47$, this becomes strictly positive, which is the condition that (C 3) is positive.Obviously $\frac{\left( c_{2}-d_{2} \right)}{\left( c_{2}-d_{2} \right)+\left( a_{2}-b_{2} \right)}$ does not exceed one in this case.

3. Note that $\frac{\left( c_{2}-d_{2} \right)}{\left( c_{2}-d_{2} \right)+\left( a_{2}-b_{2} \right)}$ is equivalent to $p_{1}^{*}$, that is, the probability that Player 1 take *H*. It is easily shown that this value is the increasing function of $a$. Furthermore, the calculation shows that $f_{2}-e_{2}>0$ if $a<0.54$. Because $f_{1}>d_{1}$ and $f_{1}>b_{1}$, $(L, D)$ constitutes a pure Nash equilibrium if $a<0.54$. Putting it differently, the mixed Nash equilibrium is the only one equilibrium in this system on the condition that $a > 0.54$.
